# Trans catheter device closure of a large azygos vein in adult patient with systemic venous collateral development after the bidirectional Glenn shunt

**DOI:** 10.34172/jcvtr.2021.14

**Published:** 2021-01-23

**Authors:** Zahra Khajali, Ata Firouzi, Pooneh Pashapour, Homa Ghaderian

**Affiliations:** ^1^Rajaie Cardiovascular Medical and Research Center, Iran University of Medical Sciences, Tehran, Iran; ^2^Interventional Cardiology Research Center, Rajaie Cardiovascular Medical and Research Center, Iran University of Medical Sciences, Tehran, Iran; ^3^Cardiovascular Research Center, Tabriz University of Medical Sciences, Tabriz, Iran

**Keywords:** Bidirectional Glenn Shunt, Azygos Vein, Transcatheter Closure

## Abstract

Superior cavopulmonary anastomosis is a type of palliative cardiac surgeries that usually done in children with cyanotic and complex congenital heart disease who have single ventricle profile. BDG shunt is staged palliation procedure for single ventricle patients who are candidates for total cavopulmonary connection (TCPC). Sometimes the surgeon misses ligating or intentionally leaves the azygos vein as a fenestration or emergency exit. This allows an abnormal flow from the superior vena cava (SVC into azygos vein). These patients can present progressive desaturation, chest tightness, progressive dyspnea, edema and shortness of breath. Therapeutic options include observation, surgical ligation and trans catheter closure. Because of high risks and extra traumas of surgery and greater chance for difficulties and the feasibility of trans catheter therapy, it is done in some centers as a method of choice.

## Introduction

Superior cavopulmonary anastomosis is a type of palliative cardiac surgeries that usually done in children with cyanotic and complex congenital heart disease who have single ventricle profile. Bidirectional Glenn (BDG) shunt is staged palliation procedure for single ventricle patients who are candidates for total cavopulmonary connection (TCPC).

Sometimes the surgeon misses ligating or intentionally leaves the azygos vein as a fenestration or emergency exit. This allows an abnormal flow from the superior vena cava (SVC into azygos vein). These patients can present progressive desaturation, chest tightness, progressive dyspnea, edema and shortness of breath. Therapeutic options include observation, surgical ligation and trans catheter closure. Because of high risks and extra traumas of surgery and greater chance for difficulties and the feasibility of trans catheter therapy, it is done in some centers as a method of choice.

## Case Presentation

**Figure 1:**
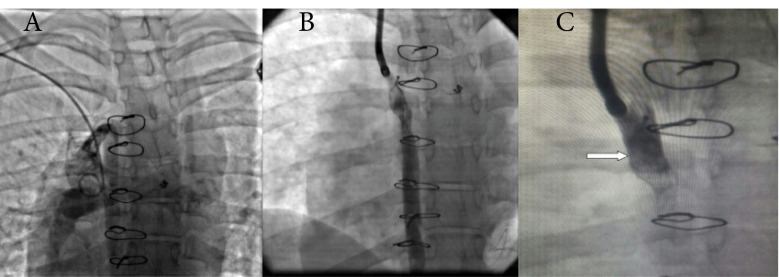
(A) SVC angiography showed a large azygos vein in frontal projection and no other pulmonary venous fistula, (B) selective azygos angiogram was taken during VSD device occlusion, (C) Azygos injection after the whole occlusion procedure, the arrow shows VSD occluder device in proper position

The present report describes a trans catheter closure of azygos vein in a patient with BDG shunt and patent azygos vein. A 24-year-old lady who admitted in hospital with gradual progressive cyanosis and dyspnea. She underwent BDG surgery 7 years ago. At first evaluation the patient was desaturated with arterial o_2_sat = 76%. she was cyanotic and had remarkable nail clubbing and lower extremities edema. In echocardiography she had Dextrocardia, criss cross heart, small right ventricle with dominant left ventricle, transposed great artery and severe valvular and subvalvular stenosis. Glenn shunt was patent with stagnant flow. For better evaluation of pulmonary artery pressure and finding the etiology of progressive cyanosis cardiac catheterization performed. In Glenn shunt injection we found large azygos vein and no other pulmonary AV fistula or venous fistula (Figure 1A) by blaming the significant runoff, the pulmonary flow to the azygos vein, we decided to close this vein. In addition due to mild elevated pulmonary artery pressure (mean of PAP = 16 mm Hg) and systemic ventricular dysfunction, the patient was not a suitable candidate for Fontan surgery.

Cardiac catheterization was performed with general and local anesthesia and 8 French catheters advanced into internal jugular vein. As soon as the intravascular access was secured a bolus injection of heparin was administered at a dose of 100 u/kg. The hemodynamic of patient monitored and X ray angiographic images of SVC and pulmonary artery were acquired after bolus injection of radiopaque media to assess the morphology of the pulmonary artery and azygos vein and its junction with the SVC. The potential site of closure was determined and marked with angiography. The diameter of azygos vein and the SVC were measured then diameter of the device was selected. Angiography of SVC was acquired and sizing balloon occlusion test performed before device deployment. The hemodynamic data including systemic saturation, azygos and SVC pressure was acquired to confirm the effectiveness of occlusion. After 15-minute occlusion test no increase in pressure occurred and systemic saturation increased from 76% to 83%, so muscular VSD septal occluder, occlutech 12 mm, was employed at the site of connection (Figure 1 B) immediately the SVC angiography was repeated for suitable position of device and successful occlusion of azygos vein (Figure 1 C).

## Discussion

Bidirectional Glenn shunt (BDG) procedure is a palliative surgery for some complex cyanotic heart disease. Some patients demonstrated progressive desaturation after BDG that can be due to venous collaterals with wide range of incidence (17%-31%) in some reports.^1,2^

There are three roots for collateral direction from superior vena cava to inferior vena cava (anterior, middle and posterior veins). Anterior and middle vein collaterals are small or moderate in size and can left safely without intervention but collaterals via posterior veins (azygos, hemiazygos and lumbar veins) are usually large and can lead to significant desaturation and cyanosis by growth.^3,4^

There are some reports on the use of devices such as coil, septal or ductal occluder to eliminate these shunts in pediatric and adolescent group.

Masura et al^5^ reported on cohort study of 45 patients with median age of 8 years with percutaneous management of cyanosis. The shunts were mostly from fenestration and all were closed by Amplatzer septal occluders. Venous collaterals were closed by Amplatzer vascular plugs and lateral tunnel leaks were closed by Amplatzer PFO occluders which was a novel use of this device for lateral tunnel leaks.

McElhinney et al^3^ reported study of 55patients with median age of 1.4 years with collateral channels after BDG and successful coil embolization and increase in SaO_2_ of 16%.Lu M. et al^6^ reported on cohort study of nine patients with progressive cyanosis after BDG and median age of 9 years with successful trans catheter occlusion of the azygos/hemiazygosvein. Total 3 hemiazygos veins and 7 azygos veins occluded. Coils were used in 4 of the procedure, PDA occluders in 3, ASD occluder in 2 and PDA occluder with coil in one procedure.

This study focused on azygos/hemiazygos veins occlusion and used different type of devices. The device for procedure depends on the severity of the shunt, the size of the lumen of the azygos/ hemiazygos vein and the candidate location for occlusion.

Our case report is a 24-year-old lady with history of complex congenital heart disease (dextrocardia, crisscross heart, TGA, severe pulmonary valve stenosis) and BDG 7 years ago however she was not suitable case for Fontan procedure and regarding to progressive cyanosis and dyspnea we focused on the closure of azygos/hemiazygos vein which had been left open intentionally during BDG procedure. Because the lumen size of this vein was large, we used muscular septal VSD occluder device 12mm with successful result without complication and shunt residue.

Our study is the first report to use VSD occluder device for closure of this venous channel in adult patient. Short term follows up with CT angiography demonstrated the good position of device and decrease in patient cyanosis.

## Conclusion

This is a report of a rare case of functional single ventricle that is not eligible for Fontan procedure and for functional capacity improvement we used a VSD occlude device for closure of azygos vein with successful result and no complication.

## Competing interests

The authors stated that they had no conflict of interest.

## Ethical approval

The authors certify that they have obtained all appropriate patient consent forms. In the form the patient has given her consent for her images and other clinical information to be reported in the journal. The patient understands that her name and initials will not be published.The patient gave written informed consent for the intervention.
